# Influence of pH, competing ions, and salinity on the sorption of strontium and cobalt onto biogenic hydroxyapatite

**DOI:** 10.1038/srep23361

**Published:** 2016-03-18

**Authors:** Stephanie Handley-Sidhu, Thomas K. Mullan, Quentin Grail, Malek Albadarneh, Toshihiko Ohnuki, Lynne E. Macaskie

**Affiliations:** 1 Schools of Geography, Earth and Environmental Sciences, The University of Birmingham, Edgbaston, Birmingham, B15 2TT, U.K; 2 Civil and Environmental Engineering, University of Strathclyde, Glasgow, G1 1XQ, U.K; 3 Advanced Science Research Center, Japan Atomic Energy Agency, Tokai, Ibraki, Japan; 4 School of Biosciences, The University of Birmingham

## Abstract

Anthropogenic radionuclides contaminate a range of environments as a result of nuclear activities, for example, leakage from waste storage tanks/ponds (e.g. Hanford, USA or Sellafield sites, UK) or as a result of large scale nuclear accidents (e.g. Chernobyl, Ukraine or Fukushima, Japan). One of the most widely applied remediation techniques for contaminated waters is the use of sorbent materials (e.g. zeolites and apatites). However, a key problem at nuclear contaminated sites is the remediation of radionuclides from complex chemical environments. In this study, biogenic hydroxyapatite (BHAP) produced by *Serratia sp.* bacteria was investigated for its potential to remediate surrogate radionuclides (Sr^2+^ and Co^2+^) from environmentally relevant waters by varying pH, salinity and the type and concentration of cations present. The sorption capacity of the BHAP for both Sr^2+^ and Co^2+^ was higher than for a synthetically produced hydroxyapatite (HAP) in the solutions tested. BHAP also compared favorably against a natural zeolite (as used in industrial decontamination) for Sr^2+^ and Co^2+^ uptake from saline waters. Results confirm that hydroxyapatite minerals of high surface area and amorphous calcium phosphate content, typical for biogenic sources, are suitable restoration or reactive barrier materials for the remediation of complex contaminated environments or wastewaters.

At the Fukushima Daiichi nuclear power plant (FDNPP) the removal of Sr and Cs radioisotopes is required from surface, ground and sea-water environments. For example, highly contaminated seawater from the FDNPP harbor is seeping through the harbor wall and contaminating groundwater. Activity estimates of radionuclides released from FDNPP to stagnant surface waters were around 288 PBq for ^134,137^Cs and 8.56 PBq for ^90^Sr, while direct releases to ocean were estimated to be 3.5 PBq for ^134,137^Cs and 52 GBq for ^90^Sr^1^. Other radionuclides released from the FDNPP were of less concern because they either have much shorter half-lives or were released in much lower quantities, although activity releases of ^60^Co (10.1 GBq) directly to seawater were in the same order of magnitude as ^90^Sr releases^1^. Cobalt-60 has also been identified as a significant environmental contaminant, alongside ^137^Cs and ^90^Sr, at nuclear sites, such as the Mayak Production Association (MPA) in Russia^2^ and recent research has shown that ionic strength and salinity is a major factor at mobilizing sediment bound Co and Sr at nuclear contaminated sites^2^,^3^,^4^.

One of the most widely applied remediation techniques for contaminated waters at both nuclear and non-nuclear sites is the use of sorbent mineral materials (e.g. zeolites and apatites). A wide range of zeolites are available with sorption properties dependent on their chemical composition and structures. Synthetic zeolites were originally used to clean up contaminated water after the Three Mile Island nuclear accident in the USA^5^, while large amounts of natural zeolites have recently been used at the FDNPP to immobilize contaminants in surface, subsurface and saline waters^6^. A natural zeolite, clinoptilolite, with a high Cs^+^ and Sr^2+^ sorption capacity, is currently used at the Site Ion Exchange Effluent Treatment Plant (SIXEP) at Sellafield Ltd, UK to clean up wastewaters^7^ and is used in this study as a comparison material.

Apatites, such as hydroxyapatite (HAP; Ca_10_(PO_4_)_6_(OH)_2_), have suitable properties for the immobilization of radionuclides, toxic metals, actinides and lanthanides^8^,^9^. The Ca^2+^ ions are located in two distinct crystallographic sites (Ca1 and Ca2) which provide exchange sites for a wide range of divalent (as well as some mono- and tri-valent) cations^10^. Additionally, amorphous grain boundaries within the polycrystalline HAP structure provide further sorption sites^11^.

Different cations display different site preferences: Sr^2+^ can substitute at the Ca1 and Ca2 sites^10^ but may also adsorb to the amorphous grain boundaries; while Co^2+^ is mainly incorporated into the amorphous phase^11^. In general, cations show a higher affinity towards HAP if they have the same charge and a similar ionic radius to the parent Ca^2+^ cation^12^. Cesium is a problematic radionuclide at the FDNPP but its sorption onto HAP is very low which can be attributed to its single charge and much larger ionic radius (0.17 nm in solution) when compared to Ca^2+^ (0.10 nm)^13^. Apatite permeable reactive barriers have been successfully trialed at the Hanford nuclear site, USA, to limit the flow of contaminants, primarily ^90^Sr, in groundwater moving towards the Columbia River^14^. More recently, apatites are being tested as a potential remediation material at the FDNPP site^15^.

Reduced capacity for the removal of targeted ions in the presence of competing ions has been identified as one of the major weaknesses of sorption/ion exchange materials^7^. Na^+^, Mg^2+^ and Ca^2+^ are common ions in natural waters and have high to moderate affinities towards HAP and zeolite materials^10^,^16^. Commercially available HAP is generally prepared via chemical synthesis and there has been widespread interest in developing low cost alternatives from bone or eggshell wastes^17^,^18^, as well as via bacterial synthesis^19^ , which can be achieved at the expense of inorganic phosphate sourced from wastewater^20^ giving a potentially low cost and environmentally friendly alternative. Alternatively, microbial mineral precipitation technologies could be utilized in the subsurface to capture radionuclide contaminants^21^,^22^,^23^,^24^. Recent research has shown that bacterially produced hydroxyapatite (BHAP) has superior sorption properties to synthetic HAP for removal of Sr^2+^ and Co^2+^ due to its amorphous content, small crystallite size and high surface area but its potential has only been evaluated in simple aqueous solutions and groundwater^11^,^25^,^26^. Hence, this study compares the potential suitability of HAP and BHAP to clean up complex waters (under varying concentrations of competing cations, salinity and pH conditions) by investigating the sorption of radionuclide surrogate metals (Sr^2+^ and Co^2+^). These materials were also compared against a natural zeolite (clinoptilolite, similar to that used at the SIXEP plant UK, since 1985^27^) to determine the suitability of materials for cleaning up saline contaminated environments, such as those found at the FDNPP.

## Results and Discussion

### Material characterization

Powder XRD analysis confirmed that the samples of BHAP and HAP were characterized as amorphous and crystalline hydroxyapatite materials, respectively (Supplementary Fig. S1 and S2). Powder XRD analysis also confirmed the identity of the clinoptilolite (general formula: KNa_2_Ca_2_(Si_29_Al_7_)O_72_.24H_2_O; Supplementary Fig. S3) and the minor metal impurities were determined by XRF (Supplementary Table S1). BHAP had a >4-fold larger specific surface area (SSA: 94 ± 4.8 m^2^/g) and smaller crystallite size (54 nm) as compared to HAP (21 ± 0.17 m^2^/g and 97 nm); these properties were previously shown to improve the sorption capacity of materials for radionuclides (i.e. Sr and Co)^11^, as well as lanthanides (Eu)^9^,^10^,^11^, transuranic elements (Cm)^8^,^28^ and cationic dyes^29^. The materials used in this study were not heat treated and contained ~30% wt dried organic content. This quantity of organic material has been previously shown to sorb Co^2+^ and Sr^2+^ by 26% and 17% of the total dissolved metals, respectively^11^.

### Effect of competing cations

**Figure 1:**
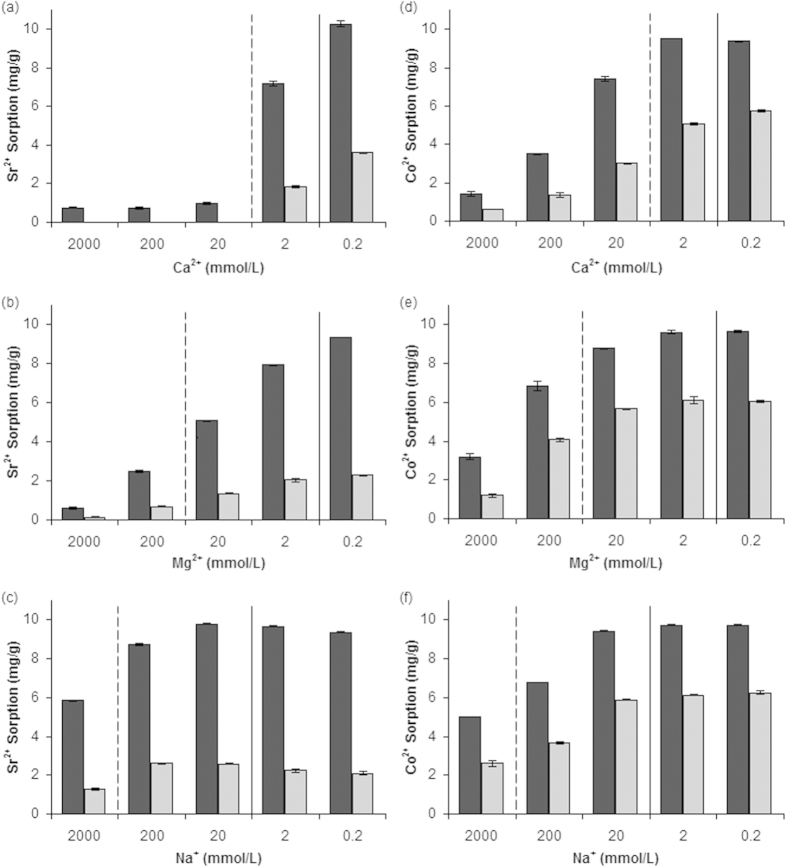
Influence of competing ion concentrations on Sr^2+^ (a–c) and Co^2+^ (d–f) sorption for BHAP (dark grey) and HAP (light grey). Error bars represent ± one standard deviation of three replicates. Dashed lines indicate approximate average concentration of competing ions in seawater; filled lines represent approximate concentrations of ions in Fukushima groundwater and Sellafield waste water (Table 1).

Although groundwater contains many other constituents (inorganic anions and organics), since the isoelectric point of apatite is pH 6^30^ and apatites are known to buffer water to more neutral conditions^31^, it follows that under conditions in most natural waters, apatites have a net negative charge and will attract cations and repel anions. For example, apatite has the ability to load high levels (20–30 wt %) of U in the presence of high background nitrate anion concentrations^32^. The concentrations of Sr^2+^ and Co^2+^ used in this study are higher than those at most radioactive contaminated sites and are more comparable with metal concentrations released from mining contamination, such as acid mine drainage^31^. However, apatites have proven ability to remediate at contaminants at environmentally relevant radionuclide concentrations^14^,^15^. The influences of competing cations on the sorption efficiencies of BHAP and HAP were investigated (Fig. 1). Between Ca^2+^ concentrations of 0.2 and 20 mmol/L Sr^2+^ sorption was reduced substantially from 10 to 0.98 mg/g for BHAP and from 3.6 to 0.021 mg/g for HAP (Fig. 1a). However, with further increases of Ca^2+^ (from 20 to 2000 mmol/L) there was no additional impact on sorption, remaining between 0.76 and 0.98 mg/g for BHAP and <0.020 mg/g for HAP. There was a much steadier decline in Sr^2+^ sorption with Mg^2+^ as the competing ion (Fig. 1b); at concentrations of 0.2 and 2000 mmol/L uptake values were reduced from 9.4 to 0.60 mg/g for BHAP and from 2.3 to 0.14 mg/g for HAP. Strontium uptake was much less influenced by Na^+^ as a competing cation. Between Na^+^ concentrations of 0.2 and 200 mmol/L, Sr^2+^ sorption onto BHAP was between 8.7 and 9.8 mg/g, while sorption onto HAP was between 2.1 and 2.6 mg/g (Fig. 1c). Higher concentrations of Na^+^ (2000 mmol/L) reduced uptake to 5.9 mg/g and 1.3 mg/g for BHAP and HAP, respectively.

Cobalt uptake values remained fairly stable between Ca^2+^ concentrations of 0.2 and 2 mmol/L (between 9.4 and 9.5 mg/g for BHAP) but decreased slightly from 5.8 to 5.1 mg/g for HAP (Fig. 1d). Further increases of Ca^2+^ up to 2000 mmol/L reduced sorption capacities of BHAP and HAP to 1.4 mg/g and 0.61 mg/g, respectively. Mg^2+^ concentrations of between 0.2 and 2 mmol/L had no discernable impact on Co^2+^ sorption, remaining between 9.6 and 9.7 mg/g for BHAP and at 6.1 mg/g for HAP (Fig. 1e). At 20 mmol/L and above, Mg^2+^ did provide competition for Co^2+^ sorption sites, with sorption values reducing to 3.2 mg/g and 1.1 mg/g for BHAP and HAP, respectively. With Na^+^ as the competing ion, Co^2+^ sorption was fairly stable between concentrations of 0.2 and 20 mmol/L at between 9.4 and 9.7 mg/g for BHAP and between 5. 9 and 6.2 mg/g for HAP (Fig. 1f). Increasing the concentration of Na^+^ up to 2000 mmol/L caused a reduction in sorption, down to 5.0 mg/g for BHAP and 2.6 mg/g for HAP.

In all cases the nanocrystalline BHAP was a more efficient sorbent than HAP, in accordance with other studies comparing the uptake of metals by commercial HAP and BHAP^11^,^25^,^26^. Ca^2+^ (the parent ion of HAP) had the largest influence over the sorption capacity followed by Mg^2+^ and then Na^+^. Much higher concentrations of Mg^2+^ are required to reduce Sr^2+^ sorption onto apatites which can be attributed to Sr^2+^ (0.13 nm) having a closer hydrated ionic radius to Ca^2+^ (0.10 nm) than Mg^2+^ (0.070 nm)^13^. The lower affinity of Na^+^ can be explained by its single charge, however, excess Na^+^ ions are likely to block the sorption sites for Sr^2+^ and Co^2+^ ions.

Examples of Ca^2+^, Mg^2+^, and Na^+^ concentrations from different environments are shown in Table 1. All three ions investigated are present at sufficient concentrations in seawater to significantly reduce the sorption capacity of materials. For Fukushima groundwater and Sellafield wastewater (Table 1) the Ca^2+^ and Mg^2+^ concentrations may have a slight impact on Sr^2+^ sorption but any effect on Co^2+^ sorption would be minimal (Fig. 1).

### pH

**Figure 2:**
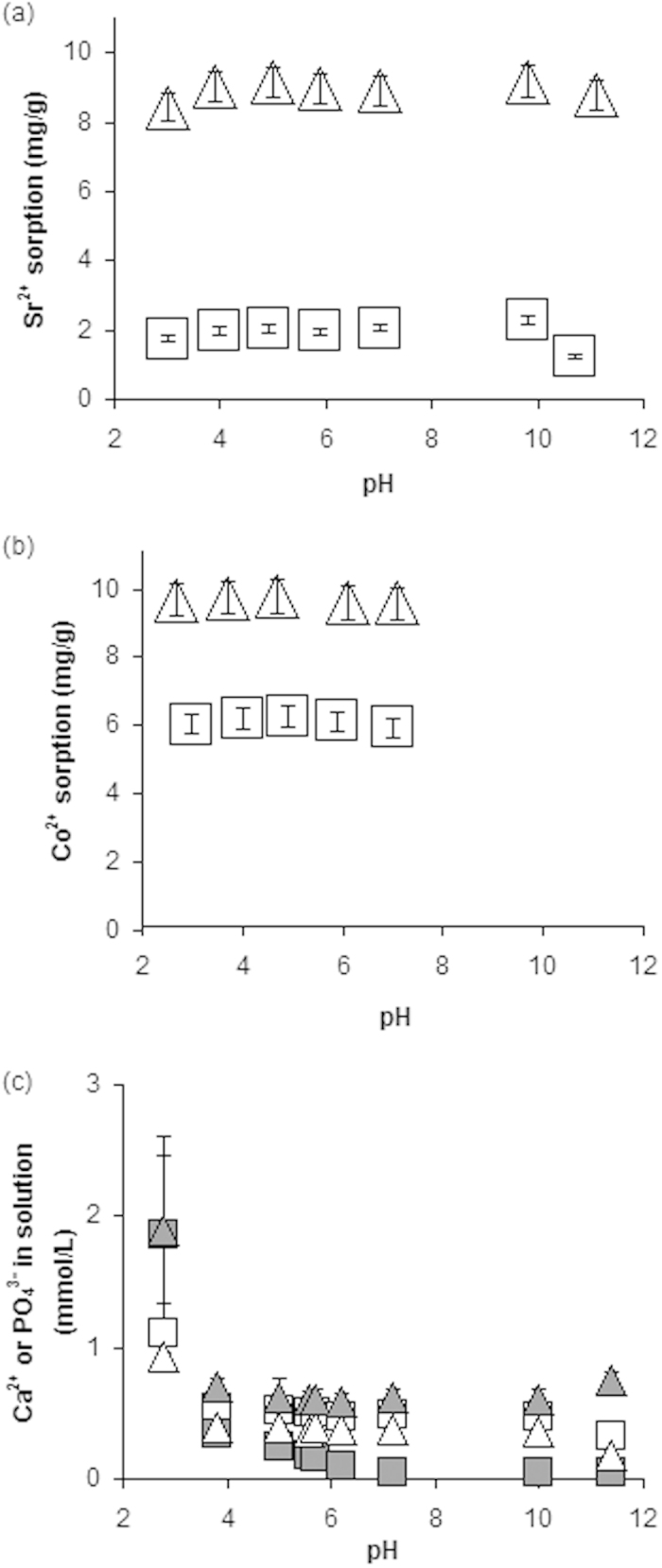
Influence of pH on the efficacy of materials (Square = HAP, Triangle = BHAP) for (**a**) Sr^2+^ sorption, (**b**) Co^2+^ sorption (pH restricted to 8 as limited by solubility of Co at higher pH values), error bars below ± 5%. The stability of materials as indicated by the input of Ca^2+^ (white) and PO_4_^3−^ (grey) to the solution phase (**c**), error bars ± 1 stdev.

The influence of pH on the sorption efficiencies of the materials was also tested. Apatite is known to buffer against large pH changes by the presence of PO_3_^4−^, OH^−^ and CO_2_^3−^ groups and has been used to remediate contamination from acid mine drainage^31^. Additionally, the organics content of the materials would aid buffering via component phosphate and carboxyl groups. The sorption of Sr^2+^ onto BHAP and HAP is shown in Fig. 2a. When mixed with solutions at pH 3 to 11 the sorption onto BHAP was fairly stable, between 8.4 and 9.2 mg/g whereas, for HAP the sorption was between 1.2 and 2.3 mg/g. Sorption of Co^2+^ (Fig. 2b) was stable from pH 3 to 7, remaining between 9.5 and 9.7 mg/g for BHAP and between 5.8 and 6.2 mg/g for HAP. At pH 8 and above dissolved Co began to hydrolyze and precipitate as the insoluble Co(OH)_2_.

The pH-dependent stability of materials, as indicated by Ca^2+^ and PO_4_^3−^ leaching into the solution phase, is shown in Fig. 2c. At most pH values, BHAP was observed to leach slightly less Ca^2+^ than HAP but more PO_4_^3−^ in accordance with it being a more calcium deficient material^11^,^33^. However, across the pH range investigated both sorbents displayed very low amounts of leaching (<2.0 mmol/L each of Ca^2+^ and PO_4_^3−^) confirming their stability and ability to buffer the pH of polluted and natural waters^31^.

### Salinity of Waters

**Figure 3:**
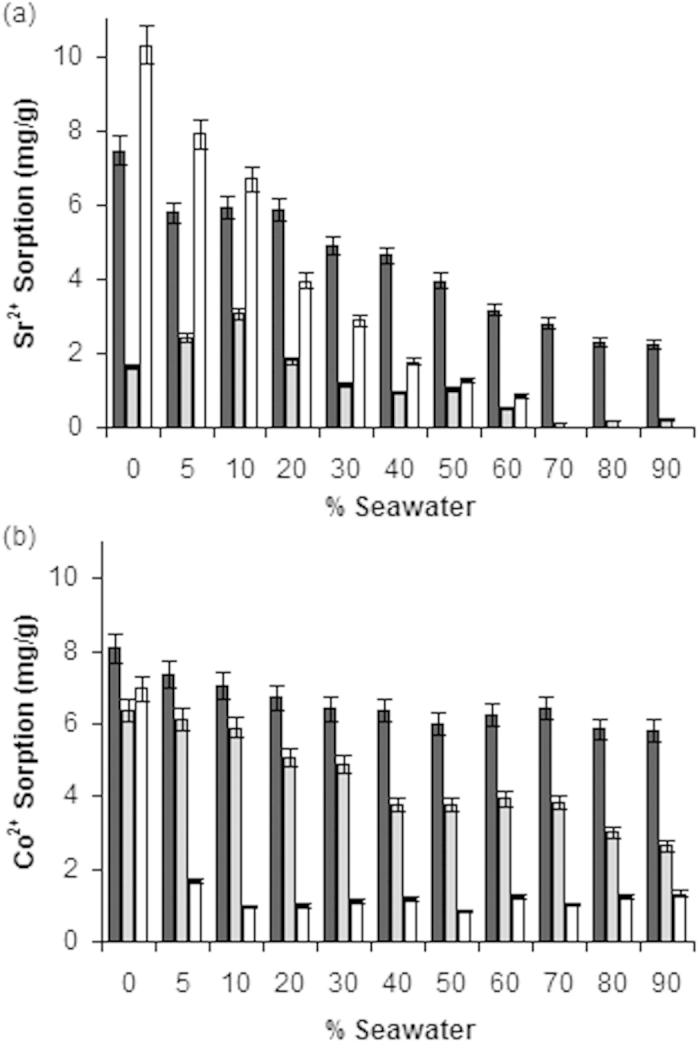
Influence of seawater concentrations on the sorption of (**a**) Sr^2+^ and (**b**) Co^2+^ onto BHAP (dark grey), HAP (light grey), and clinoptilolite (white). Error bars ± 5%.

The influence of water salinity (0 to 90% seawater sampled from Japan) on the sorption capacities of BHAP, HAP, and clinoptilolite was assessed (Fig. 3). Saline waters are a problem at many nuclear contaminated sites, such as the MPA and FDNPP. In deionized water clinoptilolite was the most efficient sorbent of Sr^2+^, removing 10.3 ± 0.51 mg/g from solution compared to 7.5 ± 0.37 mg/g and 1.6 ± 0.081 mg/g for BHAP and HAP, respectively. However, increasing salinity promoted a decrease in sorption capacity for clinoptilolite, with no uptake of Sr^2+^ at 70% seawater and above. In comparison, even at 90% seawater BHAP and HAP retained some Sr^2+^ sorption capacity at 2.3 ± 0.11 mg/g and 0.21 ± 0.015 mg/g respectively. The capacity of BHAP at 90% seawater was ten-fold higher for Sr^2+^ than that of the commercial material which may be due to its calcium deficient properties.

BHAP removed the largest amount of Co^2+^ from solution in deionized water (8.1 ± 0.40 mg/g) with uptake onto HAP (6.4 ± 0.32 mg/g) and clinoptilolite (7.0 ± 0.35 mg/g) slightly lower. The sorption efficiency of clinoptilolite fell substantially at 5% seawater to 1.7 ± 0.082 mg/g and then from 10 to 90% seawater remained fairly stable at between 0.84 and 1.3 mg/g. Cobalt sorption onto BHAP and HAP decreased more gradually with increasing salinity, reducing to 5.8 ± 0.29 mg/g and 2.6 ± 0.13 mg/g, respectively at 90% seawater. Hence, in 90% seawater, the capacity of BHAP was ~ twice that of HAP.

For all three sorbents, water salinity impacted more on Sr^2+^ sorption than for Co^2+^ sorption. The results can be partly explained by the concentration of competing ions present in seawater. Of the three competing ions investigated in this study, Ca^2+^ showed the largest influence over sorption onto hydroxyapatite, but is present in the lowest concentrations in seawater (9.4 mmol/L), while Na^+^ had the lowest impact but is present in the largest concentration (450 mmol/L)^16^. Under these concentrations, HAP and BHAP are able to retain some sorption capacity for Sr^2+^ and Co^2+^ but of the three materials BHAP is the most resilient against the effect of a more concentrated saline solution.

### Sorption isotherms

The mechanisms of metal cations retention by HAP include ion exchange, adsorption, dissolution-precipitation and substitution of Ca^2+^ ions in mineral structure^34^. Isotherms in this study were used as empirical equations rather than mechanistic models as the site of Sr^2+^ and Co^2+^ incorporation into BHAP has been well defined using extended X-ray absorption fine structure, with Sr^2+^ and Co^2+^ confirmed to mainly incorporate into the amorphous calcium phosphate phase^11^. Additionally from a previous study^25^ it is estimated that the organic content would be responsible for <30% of Co^2+^ or Sr^2+^ uptake with sorption to organic sites (such as carboxyl, phosphate and hydroxyl groups^35^). Sorption of Sr^2+^ and Co^2+^ onto BHAP and HAP was assessed across a range of initial concentrations with the results used to construct linear, Langmuir, and Freundlich isotherms (Supplementary Fig. 4a–f). The linear isotherms (supplementary Fig. S4a and S4d) was only able to fit sorption data for Sr^2+^ onto HAP (*R*^2^ = 0.99) and BHAP (*R*^2^ = 0.86) but was unsuitable for Co^2+^ sorption (*R*^2^ < 0.7).

The Langmuir model assumes a monolayer coverage of adsorbate on homogeneous sites but it is well known that the sorption of Sr^2+^ and Co^2+^ occurs via several sites including those at the surface and also within the HAP crystal. The Langmuir isotherm (supplementary Fig. S4b and S4e) did not describe the sorption of Sr^2+^ onto HAP well (*R*^2^ = 0.73), however, it fitted the sorption of Sr^2+^ onto BHAP and the sorption of Co^2+^ (*R*^2^ > 0.95) onto HAP and BHAP. The calculated maximum monolayer capacity of the sorbent (*Q*_max_; Table 2) showed that BHAP (47 mg/g for Sr^2+^, 62 mg/g for Co^2+^) has a larger maximum sorption capacity than HAP (33 mg/g for Sr^2+^, 14 mg/g for Co^2+^). The *Q*_max_ for Sr^2+^ sorption onto HAP is overestimated due to poor model fit. The separation factor (*R*_L_) was between zero and one (Table 2) for Sr^2+^ and Co^2+^ sorption onto both BHAP and HAP, indicating the favourable nature of the sorption process^36^, *R*_L_ also tended towards zero at higher initial concentrations (Table 2) showing a low degree of reversibility for Sr^2+^ and Co^2+^ sorption onto BHAP and HAP, as also shown experimentally^11^.

In contrast the Freundlich isotherm assumes adsorption to heterogeneous sites of different affinities. The Freundlich isotherm was the only model for which R^2^ > 0.95 for each sorbent. Values of 1/n (Table 3) for Sr^2+^ sorption were smaller for BHAP (0.390) than for HAP (0.566) indicating a more heterogeneous surface for BHAP, corresponding to its more amorphous nature. However, for Co^2+^, 1/n values suggested that HAP (0.130) had a more heterogeneous surface than BHAP (0.383). This may indicate that the Freundlich isotherm is not actually appropriate for describing Co^2+^ sorption in this study (the linearized Freundlich is known for its insensitivity, and so a good fit to experimental data does not necessarily mean the model is suitable)^37^. The KF values are only comparable for equal values of 1/n and, therefore, did not provide any further useful information^38^.

## Conclusion

In summary, the sorption of Sr^2+^ and Co^2+^ onto BHAP was higher than HAP under all solutions tested. BHAP sorption was least influenced by the salinity of seawater and the concentration of competing cations. Sorption was pH-independent due to buffering and materials were resistant to leaching within the pH range of most natural and contaminated waters. BHAP compared favorably against a natural zeolite (clinoptilolite – SIXEP material which is known for its high Sr^2+^ radionuclide sorption capacity) which confirms its suitability for aiding the clean up saline contaminated environments, such as those found at the FDNPP or MPA.

The advantageous sorption behavior of BHAP outlined in this study confirms the idealized properties (high SSA, small crystallite size, amorphous structure, calcium deficiency) of this material for the immobilization and removal of divalent radionuclides from complex waters. Therefore, BHAP has the potential both to be used in a permeable reactive barrier, but also as a model material by which alternative sources of HAP (e.g. from waste products such as bone meal and prepared from egg shells) could be modified to optimize their performance, for example via biomimetics based on incorporation of bacterial polymers. Additionally, *Serratia* sp. (BHAP producing bacteria) could be pumped into contaminated subsurface environments or the natural subsurface bacteria could be stimulated to precipitate metal phosphates which can immobilize aqueous radionuclides^29^.

## Methods

### Materials

Biogenic hydroxyapatite (BHAP) was manufactured using a *Serratia* sp. (NCIMB 40259). Four flasks containing 1 L of 0.1 mol/L AMPSO buffer (pH 9.2) and frozen biomass *Serratia sp*. (OD_600_ = 1.0 mg dry biomass/mL) were inoculated daily with 2 mmol/L calcium chloride, 2 mmol/L trisodium citrate and 5 mmol/L glycerol 2-phosphate. Flasks were incubated (30 °C) and shaken (100 rpm). After 8 days the BHAP was harvested (approximately 10 g) by centrifugation, oven dried (50 °C), then manually ground and sieved to <105 μm.

The performance of the BHAP was assessed by comparing it with a previously characterized reference sample of commercially available nano-sized hydroxyapatite (HAP; Sigma-Aldrich; Part number: 677418). Sorption capacity of BHAP and HAP in saline waters was also compared with to a natural SIXEP clinoptilolite.

### Characterization

Materials were characterized as per Handley-Sidhu *et al*.^11^,^25^. Samples of the materials were analyzed by X-ray diffraction (XRD; Bruker D8 Advanced X-ray diffractometer; Cu K*α* radiation). Crystallite sizes for BHAP and HAP were calculated from the characteristic peak at 2*θ* = 26° using the Scherrer equation^39^. Specific surface area (SSA) of the BHAP and HAP was determined using a BET surface area analyzer (Beckman Couter, SA 3100). The clinoptilolite was also characterized by XRD and X-ray fluorescence (XRF).

### Sorption experiments

Solutions of Sr and Co (stable isotopes) were prepared using SrCl_2_ and CoCl_2_ salts and deionised water (MQ water, ≤18.2 MΩ/cm). The sorption of Sr^2+^ and Co^2+^ onto BHAP and HAP was measured in deionized water using a range of initial solution concentrations from 0.034 to 12 mmol/L Sr^2+^ or from 0.084 to 18 mmol/L Co^2+^.

#### Investigating the influence of competing ions on the sorption capacity of BHAP and HAP

Individual stock solutions (5 mol/L) of CaCl_2_, MgCl_2_ and NaCl were prepared. Solutions were then diluted to give cation concentrations of 0.2, 2, 20, 200, 2000 mmol/L Ca^2+^, Mg^2+^ and Na^+^ and spiked to give a final concentration of 1 mmol/L Sr^2+^ or Co^2+^.

#### Investigating the influence of pH on the sorption properties of BHAP and HAP

Solutions were adjusted (1 mol/L solutions of NaOH and HCl) to give a pH range of 3–11 and a final concentration of 1 mmol/L of Sr^2+^ or Co^2+^. The stability of the materials were assessed through measuring the amount of Ca^2+^ and PO_4_^3−^ released into solution by ion chromatography (Dionex, ICS-1100).

#### Investigating the influence of seawater salinity on sorption capacity of BHAP, HAP and clinoptilolite

Seawater was sampled from Ibaraki, Japan (36°30.04′N, 142°00.09′E; 31/10/2011) at a sampling depth of 10 m, filtered (0.45 μm), and stored at room temperature in a polypropylene container. The seawater was diluted to give concentrations of between 5 to 90% seawater and spiked to give a final concentration of 1 mmol/L Sr^2+^ or Co^2+^.

All sorption experiments were carried out in triplicate. Accurately weighed masses (~0.01 g) of materials were placed in polypropylene vials and an aliquot (1.5 mL) of the appropriate solution was added. The vials were immediately positioned vertically on an orbital shaker (150 rpm) at room temperature for 24 hrs. Samples were harvested by centrifugation (16,000 g; 30 min; Sigma 1–14) and the supernatant analyzed by ion chromatography, inductively coupled plasma mass spectroscopy (ICP-MS; Agilent 7500ceor) or atomic absorption spectroscopy (AAS; Perkin Elmer, AAnalyst 300) using solution matched standards and internal reference standard.

## Additional Information

**How to cite this article**: Handley-Sidhu, S. *et al*. Influence of pH, competing ions, and salinity on the sorption of strontium and cobalt onto biogenic hydroxyapatite. *Sci. Rep.* **6**, 23361; doi: 10.1038/srep23361 (2016).

## Supplementary Material

Supplementary Information

## Figures and Tables

**Table 1:** The concentration of cations (Na^+^, Mg^2+^ and Ca^2+^) in different environmental samples (average global river water^40^; seawater from Imari Bay, Japan^16^; FDNPP groundwater^15^; and Magnox waste storage tank liquor from Sellafield, UK^3^).

**Ion**	**Concentration (mmol/L)**			
	**Seawater**	**River water**	**Fukushima groundwater**	**Sellafield tank liquor**
Na^+^	450	0.31	2.1	6.6
Mg^2+^	52	0.15	0.86	0.39
Ca^2+^	9.4	0.37	0.70	0.27

**Table 2:** Langmuir isotherm parameters.

**Metal**	**Sorbent**	**Intercept**	**Slope**	***R***^**2**^	***Q***_**max**_**(mg/g)**	***R***_**L**_	
						**Lowest** ***C***_**0**_	**Highest** ***C***_**0**_
Sr^2+^	HAP	9.4	0.030	0.73	33	0.99	0.23
	BHAP	1.3	0.021	0.96	47	0.96	0.058
Co^2+^	HAP	1.5	0.074	1.0	14	0.78	0.019
	BHAP	0.42	0.016	0.99	62	0.82	0.024

**Table 3:** Freundlich isotherm parameters.

**Sorbate**	**Sorbent**	**Intercept**	**Slope (1/*****n***)	***R***^**2**^	***K***_**F**_
Sr^2+^	HAP	−0.276	0.566	0.970	0.530
	BHAP	0.566	0.390	0.998	3.68
Co^2+^	HAP	0.745	0.130	0.987	5.56
	BHAP	0.804	0.383	0.959	6.37

## References

[b1] Povinec P. P., Hirose K. & Aoyama M. Radioactivity impact on the environment in Fukushima accident 103–130 (Elsevier Inc, 2013).

[b2] Standring W. J. F., Oughton D. H. & Salbu B. Potential remobilization of Cs-137, Co-60, Tc-99 and Sr-90 from contaminated Mayak sediments river and estuary environments. Environ. Sci. Technol. 36, 2330–2337 (2002). 12075786 10.1021/es0103187

[b3] Wallace S. H. . Effect of groundwater pH and ionic strength on strontium sorption in aquifer sediments: Implications for Sr-90 mobility at contaminated nuclear sites. Appl. Geochem. 27, 1482–1491 (2012).

[b4] Eagling J., Worsfold P. J., Blake W. H. & Keith-Roach M. J. Fate of Sr-90 and U(VI) in Dounreay sediments following saline inundation and erosion. Chemosphere 92, 911–917 (2013). 23541149 10.1016/j.chemosphere.2013.02.059

[b5] Dyer A. Applications of natural zeolites in the treatment of nuclear wastes and fall-out In Environmental mineralogy: microbial interactions, anthropogenic influences, contaminated land and waste management (Eds Cotter-Howells J. D., Campbell L. S., Valsami-Jones E., Batchelder M.) 319–368 (Mineralogical Society Series 9, 2000).

[b6] Farid O., Shih K., Lee W. E. & Yamana H. Fukushima: The current situation and future plans In Radioactive waste management and contaminated site clean-up (eds Lee William E., Ojovan Michael I., & Jantzen Carol M.) 744–776e (Woodhead Publishing, 2013).

[b7] IAEA. Application of Ion Exchange Processes for the Treatment of Radioactive Waste and Management of Spent Ion Exchangers. Technical Reports Series No. 408. (International Atomic Energy Agency, 1994).

[b8] Oelkers E. H. & Montel J. M. Phosphates and nuclear waste storage. Elements 4, 113–116 (2008).

[b9] Rakovan J. F. & Pasteris J. D. A., Technological gem: Materials, medical, and environmental mineralogy of apatite. Elements 11, 195–200 (2015).

[b10] Hughes J. M. & Rakovan J. F. Structurally robust, chemically diverse: apatite and apatite supergroup minerals. Elements 11, 165–170 (2015).

[b11] Handley-Sidhu S. . Bacterially produced calcium phosphate nanobiominerals: sorption capacity, site preferences, and stability of captured radionuclides. Environ. Sci. Technol. 48, 6891–6898 (2014). 24823240 10.1021/es500734n

[b12] Shimabayashi S., Tamura C. & Nakagaki M. Adsorption of mono-valent and divalent metal-cations on hydroxyapatite in water. Chem. Pharm. Bull. 29, 2116–2122 (1981).

[b13] Marcus Y. Ionic-radii in aqueous-solutions. Chem. Rev. 88, 1475–1498, (1988).

[b14] Vermeul V. R. . An injectable apatite permeable reactive barrier for *in situ* sr-90 immobilization. Ground Water Monit. R. 34, 29–42 (2014).

[b15] TEPCO. About the completion of the countermeasure work to address the downward flow of strontium present in the soil in relation to the leak in H4 area tank (2014). Available at: http://www.tepco.co.jp/en/nu/fukushima-np/handouts/2014/images/handouts_140911_05-e.pdf (Accessed: 1st February 2015).

[b16] Wajima T. Ion exchange properties of Japanese natural zeolites in seawater. Anal. Sci. 29, 139–141 (2013). 23303099 10.2116/analsci.29.139

[b17] Smiciklas I. . Resource recovery of animal bones: Study on sorptive properties and mechanism for Sr^2+^ ions. J. Nucl. Mater. 400, 15–24 (2010).

[b18] Meski S., Ziani S. & Khireddine H. Removal of lead ions by hydroxyapatite prepared from the egg shell. J. Chem. Eng. Data 55, 3923–3928 (2010).

[b19] Macaskie L. E. . A novel non line-of-sight method for coating hydroxyapatite onto the surfaces of support materials by biomineralization. J. Biotechnol. 118, 187–200, (2005). 15964651 10.1016/j.jbiotec.2005.03.006

[b20] Yong P., Macaskie L. E., Sammons R. L. & Marquis P. M. Synthesis of nanophase hydroxyapatite by a *Serratia* sp. from wastewater containing inorganic phosphate. Biotechnol. Lett. 26, 1723–1730 (2004). 15604826 10.1007/s10529-004-3744-4

[b21] Cuthbert M. O. . A field and modeling study of fractured rock permeability reduction using microbially induced calcite precipitation. Environ. Sci. Technol. 47, 13637–13643 (2013). 24147737 10.1021/es402601g

[b22] Fujita Y. . Stimulation of microbial urea hydrolysis in groundwater to enhance calcite precipitation. Environ. Sci. Technol. 42, 3025–32 (2008) 18497161 10.1021/es702643g

[b23] Martin D., Dodds K., Ngwenya B. T., Butler I. B. & Elphick S. C. Inhibition of Sporosarcina pasteurii under anoxic conditions: Implications for subsurface carbonate precipitation and remediation via ureolysis. Environ. Sci. Technol. 46, 8351–8355 (2012). 22774923 10.1021/es3015875

[b24] Newsome L., Morris K., Trivedi D., Bewsher A. & Lloyd J. R. Biostimulation by glycerol phosphate to precipitate recalcitrant uranium(IV) phosphate. Environ. Sci. Technol. 49, 11070–11078 (2015). 26292021 10.1021/acs.est.5b02042

[b25] Handley-Sidhu S. . Uptake of Sr^2+^ and Co^2+^ into Biogenic Hydroxyapatite: Implications for Biomineral Ion Exchange Synthesis. Environ. Sci. Technol. 45, 6985–6990 (2011). 21714547 10.1021/es2015132

[b26] Handley-Sidhu S., Renshaw J. C., Yong P., Kerley R. & Macaskie L. E. Nano-crystalline hydroxyapatite bio-mineral for the treatment of strontium from aqueous solutions. Biotechnol. Lett. 33, 79–87 (2011). 20824306 10.1007/s10529-010-0391-9

[b27] Leonard K. S., McCubbin D. & Lovett M. D. Physico-chemical characterisation of radionuclides discharged from a nuclear establishment. Sci. Total Environ. 175, 9–24 (1995).

[b28] Holliday K. . A new incorporation mechanism for trivalent actinides into bioapatite: A TRLFS and EXAFS study. Environ. Sci. Technol. 28, 3845–3851 (2012). 10.1021/la300014a 22313032

[b29] Wei W. . Fast removal of methylene blue from aqueous solution by adsorption onto poorly crystalline hydroxyapatite nanoparticles. Digest J. Nanomat. Biostruct. 19, 1343–1363 (2015).

[b30] Tarasevich Yu. I., Shkutkova E. V. & Janusz W. Sorption of Ions of Heavy Metals from Aqueous Solutionson Hydroxylapatite. J. Water Chem. Techno 34, 125–132 (2012)

[b31] Conca J. L. & Wright J. An apatite II permeable reactive barrier to remediate groundwater containing Zn, Pb and Cd. Appl. Geochem. 21, 1288–1300 (2006).

[b32] Bostick W. D., Stevenson R. J., Jarabek R. J. & Conca J. L. Use of apatite and bone char for the removal of soluble radionuclides in authentic and simulated DOE groundwater. Adv. Environ. Res. 3, 488–498 (2000).

[b33] Ledo H. M. . Microstructure and composition of biosynthetically synthesised hydroxyapatite J. Mater. Sci. Mater. Med. 19, 3419–3427 (2008). 18568391 10.1007/s10856-008-3485-3

[b34] Rosskopfova O., Galambos M. & Rajec P. Study of sorption processes of strontium on the synthetic Hydroxyapatite J. Radioanal. Nucl. Chem. 287, 715–722 (2011)

[b35] Fein J. B., Daughney C. J., Yee M. & Davis T. A. A chemical equilibrium model for metal adsorption onto bacterial surfaces. Geochimica et Cosmochimica Acta. 61, 3319–3328 (1977).

[b36] Foo K. Y. & Hameed B. H. Insights into the modeling of adsorption isotherm systems. Chem. Eng. J. 156, 2–10 (2010).

[b37] Goldberg S. Equations and Models Describing Adsorption Processes in Soils in Chemical Processes in Soils (Eds Tabatabai M. A., Sparks D. L.) 489–517 (Soil Science Society of America Book Series, 2005).

[b38] Coles C. A. & Yong R. N. Use of equilibrium and initial metal concentrations in determining Freundlich isotherms for soils and sediments. Eng. Geol. 85, 19–25 (2006).

[b39] Patterson A. L. The Scherrer formula for x-ray particle size determination. Phys. Rev. 56, 978–982 (1939).

[b40] Brezonik P. L. & Arnold W. A. Water Chemistry: An Introduction to the Chemistry of Natural and Engineered Aquatic Systems (Oxford University Press, 2011).

