# Supplementary material for: Influence of pH, competing ions, and salinity on the sorption of strontium and cobalt onto biogenic hydroxyapatite
Source: Sci Rep. 2016 Mar 18;6:23361. doi: 10.1038/srep23361 (PMC4796913; doi:10.1038/srep23361)
Supplement: Supplementary Information [file srep23361-s1.pdf]

Influence of pH, competing ions, and salinity on the sorption of strontium and cobalt onto biogenic hydroxyapatite

*Stephanie Handley-Sidhu *[1], Thomas K. Mullan[2], Quentin Grail[1], Malek Albadarneh[1], Toshihiko Ohnuki[3], and Lynne E. Macaskie[4]*

Schools of [1]Geography, Earth and Environmental Sciences and [4]Biosciences The University of Birmingham, Edgbaston, Birmingham, B15 2TT, U.K. [2]Civil and Environmental Engineering, University of Strathclyde, [3]Environmental Sciences Research, Japan Atomic Energy Agency, Tokai, Ibraki, Japan

* Corresponding author: E-mail: s-handley-sidhu@bham.ac.uk

Supplementary Information

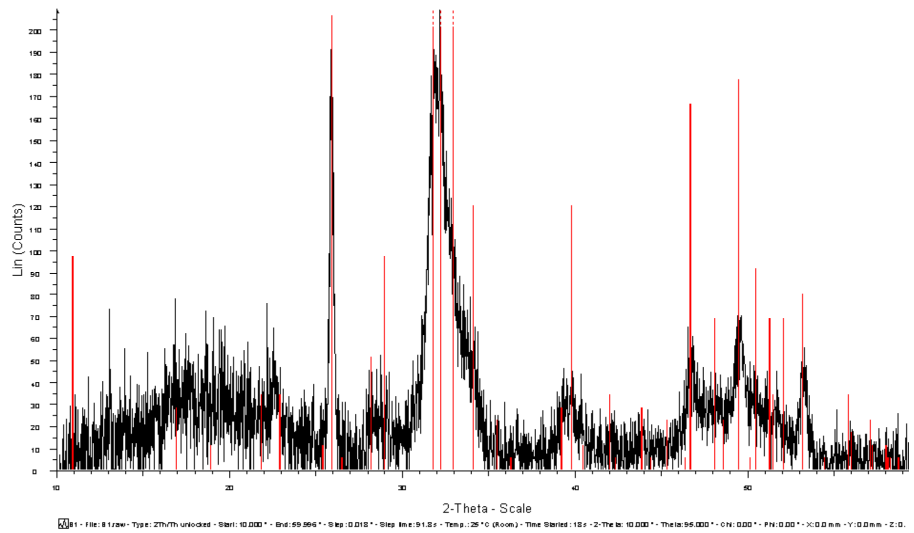

Figure S1: XRD pattern for biogenic hydroxyapatite (BHAP). Matching JCPDS database patterns 01-076-0694.

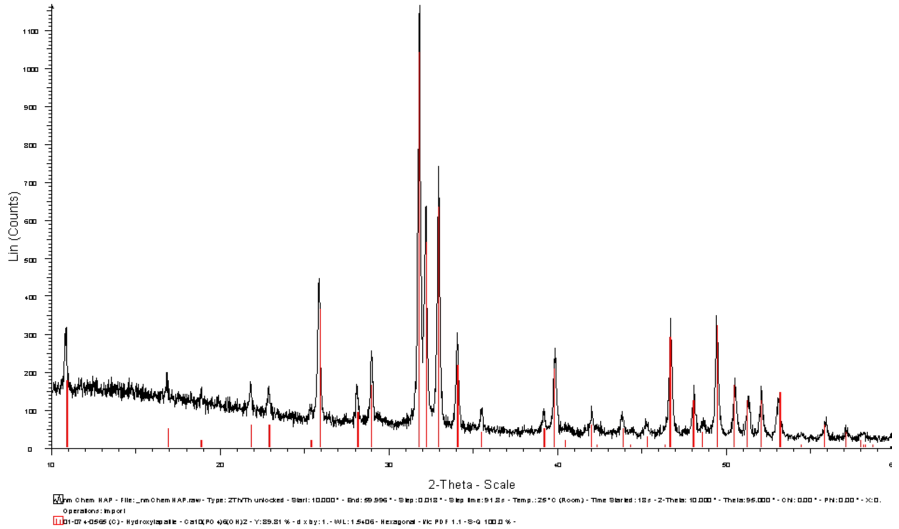

Figure S2: XRD pattern for synthetic hydroxyapatite (HAP). Matching JCPDS database 01-074-0565

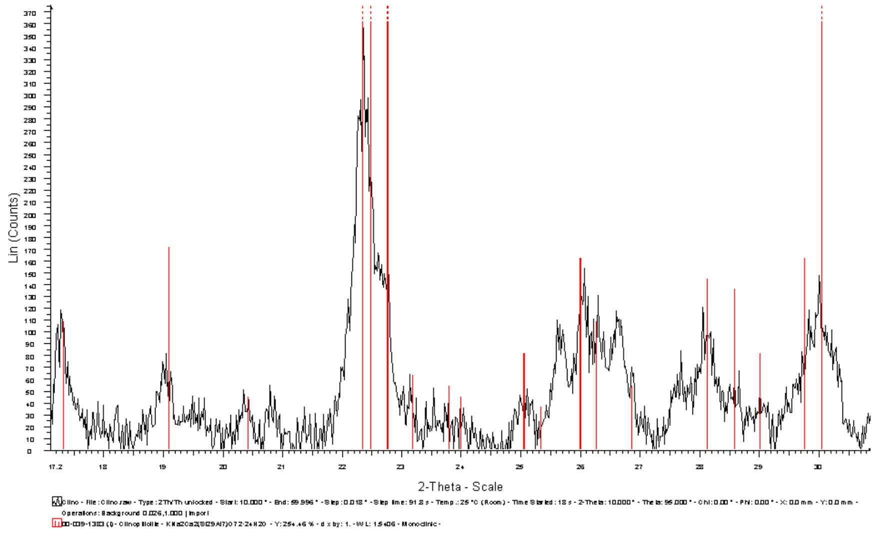

Figure S3: XRD spectrum for clinoptilolite. Matching JCPDS database pattern 00-039-1383.

Table S1: Clinoptilolite - minor metal oxide composition determined by XRF.

Elements	Sr	Fe	S	Ba	Cl	Mg	Ti	P
Composition %	1.39	1.36	0.67	0.32	0.18	0.12	0.09	0.09

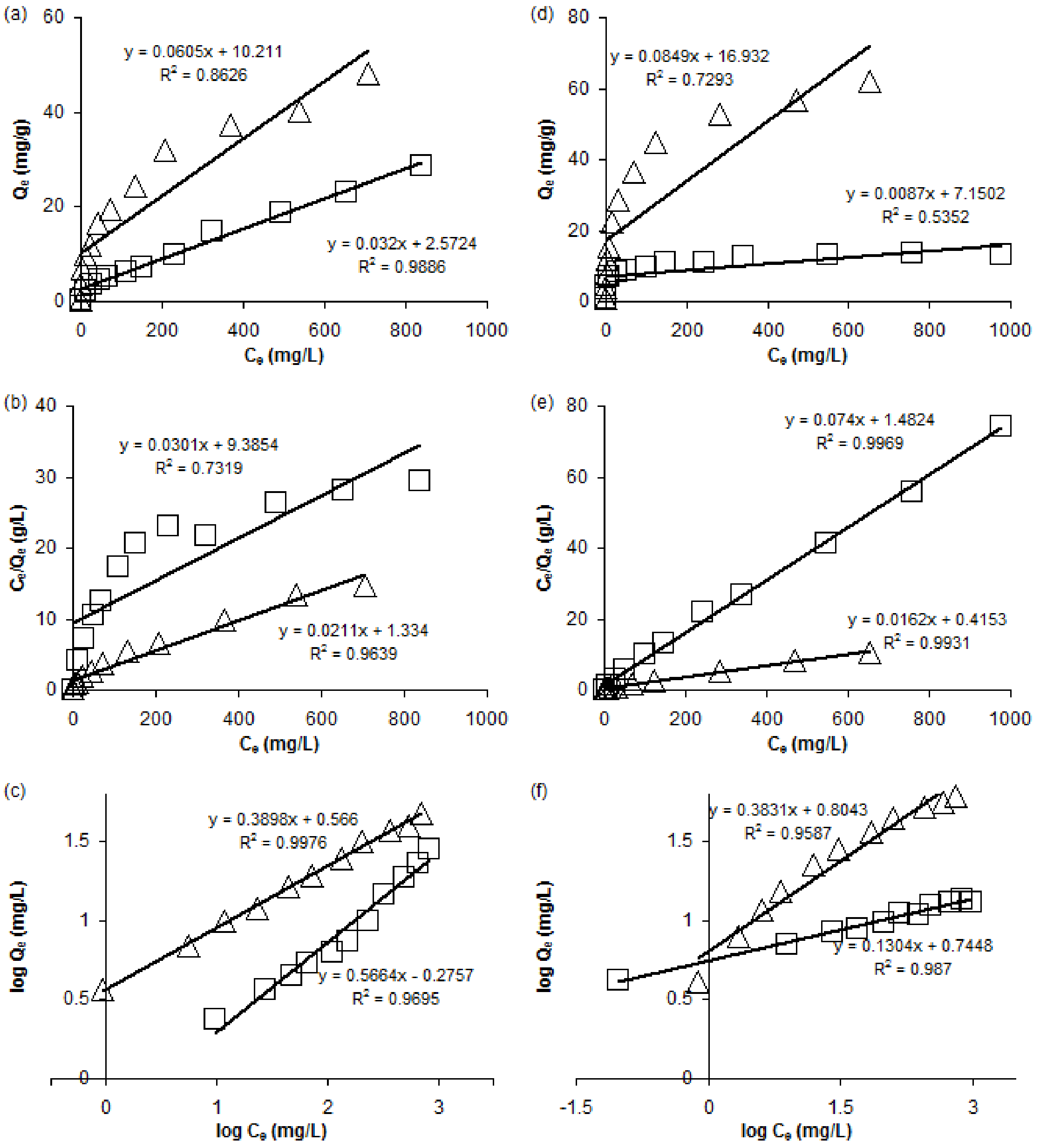

Figure S4. Sorption isotherms: (a,d) Linear; (b,e) Langmuir; and (c,f) Freundlich isotherms models for (a-c) Sr^{2+} and (d-f) Co^{2+} sorption onto BHAP (triangle) and HAP (square).

Methods for Sorption isotherms. Batch sorption experiments were performed and linear least squares regression used to determine how well the experimental data fit linear, Langmuir, and Freundlich type isotherm models.

Linear. The linear isotherm is described by equation 1 and is expressed via a plot of Q_e vs. C_e; K_d is the distribution coefficient, which provides an indication of the distribution of a sorbate between solid and solution phases (L/mg)[1,2]. **Equation 1** = $Q_e = K_d C_e$

Langmuir. A linear form of the Langmuir isotherm is given by equation 2 and expressed with a plot of C_e/Q_e vs. C_e, where Q_{max} represents the maximum monolayer capacity of the sorbent (mg/g) and K_L is a constant related to the rate of adsorption (L/mg)[3].

Equation 2 = $C_e/Q_e = C_e/Q_{max} + 1/K_L Q_{max}$

The separation factor R_L is given by equation 3, where a lower value indicates a more favorable sorption process[3]. **Equation 3** = $R_L = 1/(1+K_L C_0)$

Freundlich. A linear form of the Freundlich isotherm is given by equation 4 and expressed via a plot of log Q_e vs. log C_e where K_F is a constant related to adsorption capacity $[(mg/g)(L/mg)^{1/n}]$ and $1/n$ represents surface heterogeneity (with lower values indicating a more heterogeneous surface)[1,3]. **Equation 4** = $\log Q_e = \log K_F + (1/n) \log C_e$

References

(1) Goldberg, S. Equations and Models Describing Adsorption Processes in Soils. In *Chemical Processes in Soils*; Tabatabai, M. A., Sparks, D. L., Eds.; Soil Science Society of America: Madison, 2005, pp 489-517.

(2) Bunde, R. L.; Rosentreter, J. J.; Liszewski, M. J.; Hemming, C. H.; Welhan, J. Effects of calcium and magnesium on strontium distribution coefficients. *Environ. Geol. (Heidelberg, Ger.)* **1997**, *32*(3), 219–229.

(3) Foo, K. Y.; Hameed, B. H. Insights into the modeling of adsorption isotherm systems. *Chem. Eng. J. (Amsterdam, Neth.)* **2010**, *156* (1), 2–10.
